# Emergence of a Novel Highly Pathogenic Recombinant RNA Virus of Picornaviridae with Blood–Brain Barrier Breaching Capability in China

**DOI:** 10.3390/ani16131968

**Published:** 2026-06-25

**Authors:** Jianli Shi, Shuo Wang, Chang Liu, Yong Ying, Yongming Wang, Xiaofei Song, Lianguo Wei, Guang Zhang, Shaojian Xu, Shun Zhou, Chen Li, Jun Li

**Affiliations:** 1 Key Laboratory of Livestock and Poultry Multi-Omics of Agriculture and Reral Affairs, Shandong Research Center of Livestock and Poultry Biologicals Engineering, Institute of Animal Science and Veterinary Medicine Shandong Academy of Agricultural Sciences, Jinan 250100, China; sjl6296@163.com (J.S.); wangshuod@126.com (S.W.); liuchangsaas@163.com (C.L.); xsjcat@163.com (S.X.); 2 New Drug Evaluation Center of Shandong Academy of Pharmaceutical Sciences, Jinan 250100, China; yingyong@sdaps.cn; 3 Shandong Huahong Biological Engineering Co., Ltd., Binzhou 256000, China; wym197972@163.com; 4 Qilu Animal Health Products Co., Ltd., Jinan 250100, China; xiaofei.song@qilu-pharma.com (X.S.); lianguo.wei@qilu-pharma.com (L.W.); guang.zhang@qilu-pharma.com (G.Z.); 5 College of Veterinary Medicine, Qingdao Agricultural University, Qingdao 266109, China; zhoushun@qau.edu.cn

**Keywords:** porcine sapelovirus, recombinant, highly pathogenic, severe diarrhea, blood–brain barrier

## Abstract

Picornaviruses pose a significant threat to both human and animal health, causing many diseases in humans and swine. Porcine sapelovirus (PSV) is a globally reported enteric picornavirus commonly associated with subclinical or mild enteric infections in swine populations. Critically, unlike other neurotropic picornaviruses such as Enterovirus A71, no PSV strain has been reported to breach the blood–brain barrier (BBB). Since 2023, outbreaks of diarrhea with concurrent neurological signs like ataxia and lameness have been observed in weaned piglets across China, in particular, on a farm in Zhejiang province in 2025 with 100% morbidity and 20% mortality among the cases. Routine diagnostics ruled out common swine pathogens, but qPCR was positive for PSV. We successfully isolated three PSV strains (ZJ, FJ, SD) from affected piglets. Genetic analysis revealed that the PSV-ZJ is a novel recombinant between strains YC2011/2012 (China, 2011) and XTND/2019 (Vietnam, 2018). Pathogenicity assessment confirmed that the recombinant PSV-ZJ is highly pathogenic, causing severe diarrhea, growth retardation, and significant viral shedding via the respiratory and digestive tracts. qPCR and histopathology confirmed viral presence in intestinal and brain tissues, indicating that PSV-ZJ can cross the blood–brain barrier. This study presents the first quantitative viral load and histopathological data for a highly pathogenic recombinant PSV strain in China and emphasizes the critical role of recombination in driving viral virulence evolution, necessitating enhanced surveillance and the development of targeted preventive strategies.

## Simple Summary

A new recombinant RNA virus that infects pigs and can cross into the brain was discovered in China. This study identified the virus, showed that it causes severe disease, and found that it can break through the blood–brain barrier. These findings will help scientists and veterinarians better monitor and prevent potential outbreaks, protecting both animal health and public safety.

## 1. Introduction

Porcine sapelovirus (PSV), a member of the genus Sapelovirus (family Picornaviridae), possesses a positive-sense RNA genome with typical picornaviral organization [1,2]. The approximately 7.5 kb genome comprises a 5′untranslated region (UTR), a single large open reading frame (ORF), a 3′-UTR, and a poly (A) tail [3]. Initially identified in the United Kingdom, PSV has since been reported worldwide, with detection rates ranging from 10% to 71% [4,5,6,7,8]. Among them, one testing in Italy found that fecal pools from young growers (63/64) were found to be positive on all farms, while detection in sows (4/28) was observed on only one farm [8]. In China, surveillance since 2009 has shown infection rates of 11.2–20.4%, indicating its widespread circulation in swine herds [3,9,10,11]. However, as PSV infections often co-occur with other enteric pathogens, their clinical manifestations, such as diarrhea and encephalitis, have frequently been overlooked [12]. Unlike Enterovirus A71 within the same family, no PSV strain has previously been demonstrated to cross the BBB with direct quantification of viral RNA in brain tissue or with histopathological evidence of BBB disruption.

Since 2023, outbreaks of diarrhea in weaned piglets have been reported across major pig-producing regions of China, with some affected animals exhibiting neurological symptoms, including ataxia and forelimb lameness. Notably, a farm in Zhejiang Province experienced a severe outbreak of diarrhea in 2025, characterized by 100% morbidity and 20% mortality among weak piglets. Quantitative PCR screening of samples from affected piglets was negative for common swine enteric and systemic pathogens (including PEDV, PDCoV, PRRSV, PCV2 and CSFV) but positive for PSV. Subsequent inoculation of samples from diseased pigs onto PK-15 cells led to the isolation of three PSV strains (ZJ, FJ, and SD). An animal challenge study using the PSV-ZJ strain demonstrated that this is a novel recombinant strain capable of breaching the blood–brain barrier; the strain was linked to high morbidity and mortality during this outbreak, marking its first emergence in China.

## 2. Materials and Methods

### 2.1. Virus Isolation and Identification

PSV-positive anal swab samples were suspended in PBS, clarified by centrifugation, filtered, and inoculated onto PK-15 cells. Following incubation, cells were monitored daily for cytopathic effects (CPEs). The virus was serially passaged 10 times, purified by plaque assay, and characterized by PCR, TCID_50_ determination, one-step growth curve analysis, and transmission electron microscopy (TEM).

### 2.2. Homology and Genetic Evolution Analysis

Viral RNA was extracted, reverse-transcribed, and sequenced. Genome alignments and phylogenetic analyses were performed using MegAlign of DNASTAR 7.1 and MEGA-X 11 software, while recombination events were identified with RDP5 and SimPlot 3.5.1.

### 2.3. Pathogenicity Test in Piglets

Ten 6-day-old piglets, tested negative for PSV, PEDV, and PTV, were randomly divided into two groups (*n* = 5 each). Challenge group piglets were orally administered 15 mL of the 10th-passage PSV-ZJ virus (10^6.5^ TCID50/mL). Controls received an equivalent volume of cell culture medium. Clinical signs and body temperature were recorded daily. Body weights were recorded pre and post-challenge. Anal and nasal swabs were collected daily for viral RNA extraction and qPCR analysis of viral shedding.

One piglet was euthanized on day 8 post-infection (pi) for necropsy. Tissues (brain, lungs, intestines) were examined for gross lesions. Samples from multiple organs (heart, liver, spleen, lungs, kidneys, various gut segments, lymph nodes, thymus, brain) were collected for histopathological examination (processed by Shandong Academy of Pharmaceutical Sciences) and for viral load quantification via qPCR. Serum was collected for neutralizing antibody titer determination.

## 3. Results

### 3.1. Virus Isolation and Identification

**Figure 1:**
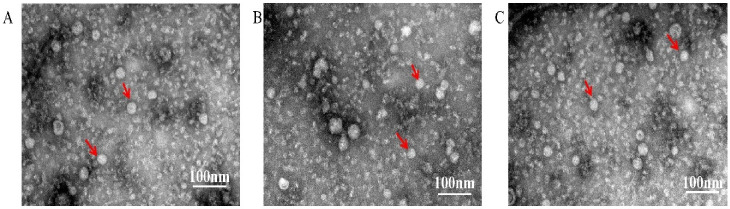
Electron microscope observation of virus isolation (250,000×). (**A**) PSV-ZJ strain electron microscope particle photo; (**B**) PSV-FJ strain electron microscope particle photo; (**C**) PSV-SD strain electron microscope particle photo.

Inoculation of PK-15 cells with sample supernatants induced slight CPE (cell rounding, brightening, partial detachment) at 24 h post-inoculation (hpi), progressing to extensive cell detachment by 48 hpi. Control cells remained unaffected. The virus was serially passaged 10 times, with stable CPE observed in each passage. Plaque purification was performed and the plaque-pured 10th-passage virus was used for the subsequent passages. PCR and CPE results of the 10th-passage virus confirmed PSV identity (Supplementary Materials). TEM revealed non-enveloped, spherical viral particles approximately 30 nm in diameter, consistent with the typical characteristics of PSV virions (Figure 1). These results confirm the isolation of a PSV strain, named PSV-ZJ. The titer of the 10th-passage virus was calculated using the Reed-Muench method, and a one-step growth curve was plotted. The virus reached a peak of 10^8.33^ TCID_50_/mL between 36 and 48 hpi. The other two isolated strains were named PSV-FJ and PSV-SD strains, with the highest titer of 10^7.5^ and 10^7.8^ TCID_50_/mL between 36 and 48 hpi.

### 3.2. Genetic Evolution and Recombination Analysis

**Figure 2:**
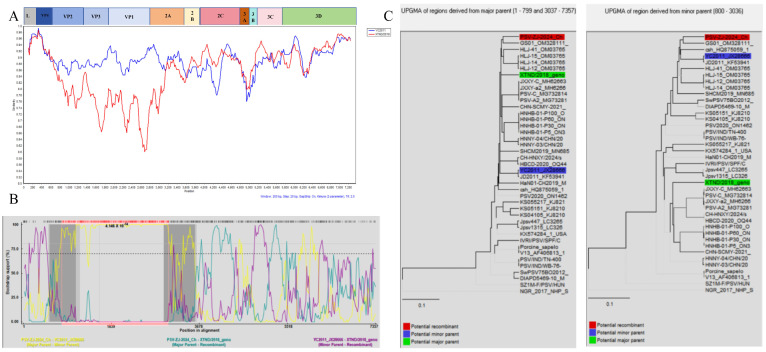
Recombination analysis of PSV–ZJ strain. (**A**) Analysis results from Simplot 3.5.1 software; (**B**,**C**) Analysis results from RDP5 software.

The complete genome length of PSV-ZJ was 7353 bp (Genbank No.PX440383), PSV-SD was 7495 bp (Genbank No.PX440381) and PSV-FJ was 7408 bp (Genbank No.PX440382). Amino acid sequence comparison with 44 reference strains showed isolates shared 90.6–99.3% identity with each other and 86.9–91.6% with recent Chinese strains, but only 76.9% with a Hungarian reference strain. Phylogenetic analysis indicated they formed a distinct clade within Chinese isolates. Recombination analysis indicated that PSV-ZJ originated from a major parental strain YC2011/2012 (GenBank No.JX286666.1, China, 2011) and a minor parental strain XTND/2019 (GenBank No.LC493088.1, Vietnam, 2018). The predicted recombination breakpoints located between the VP2 and 2A genes. This inter-regional recombination event, potentially facilitated by international trade, underscores emerging challenges for global disease control (Figure 2).

### 3.3. Pathogenicity Test Results

**Figure 3:**
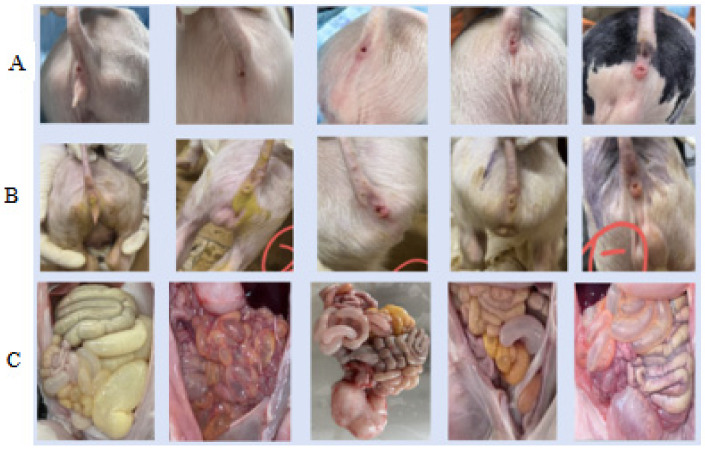
Results of clinical diarrhea and intestinal autopsy in experimental pigs post-challenge. Line (**A**) The control groups without diarrhea and ataxia. Line (**B**) Four infected piglets had diarrhea on the 6th dpc and all five piglets had diarrhea on the 7th dpc in challenged groups. Line (**C**) Both euthanized and dead piglets showed thinning of the intestinal wall and distension in challenged groups.

Piglets challenged with PSV-ZJ developed diarrhea and ataxia at 6 days post-challenge (dpc). Relative daily weight gain was significantly reduced compared with controls. One pig was euthanized at 8 dpc, 2 died at 11 dpc, and the remaining were euthanized at 13 dpc. Necropsy revealed intestinal distension and wall thinning, pulmonary congestion, and cerebral edema in infected pigs. No significant lesions were observed in control groups (Figure 3).

The mean body temperature of challenged piglets decreased progressively over the first 8 dpc (ranging from −0.23 °C to −1.0 °C), with a more pronounced body temperature by 9 dpc, suggesting the recombinant virus may manipulate host thermoregulation to favor viral replication.

Viral shedding was detected in nasal swabs from 3 dpc, with all piglets positive by 6 dpc, indicating respiratory tract infection and shedding of recombinant PSV-ZJ strain. Detection via fecal and anal swabs was positive starting at 3 dpc, and all challenged piglets tested positive by 5 dpc, confirming robust digestive tract infection and shedding. No virus was detected in control piglets throughout the study. This confirms that the recombinant PSV-ZJ strain can infect the digestive tract and be shed continuously through digestive system. Virus re-isolated from swabs was genetically identical to the challenged PSV-ZJ.

Viral load detection results showed the presence of virus in brain, stomach, all intestinal segments and mesenteric lymph nodes of challenged piglets. The highest viral loads were found in the colon with 10^8.77^ copies/g. No virus was detected in heart, liver, spleen, lungs, kidneys, or blood. Serum neutralizing antibody titers increased during infection, reaching 1:45 at necropsy. The virus was reisolated and identified from the autopsy samples, and the genome sequencing results were consistent with the PSV-ZJ strain used for challenged.

**Figure 4:**
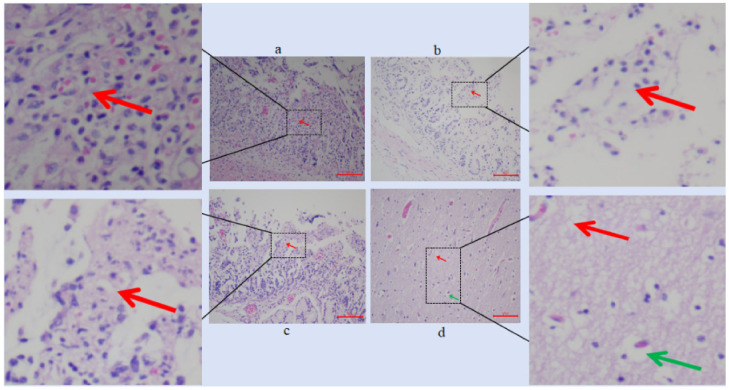
Organs pathological examination results in dead piglets. (**a**) There are villus atrophy, mucosal epithelial degeneration and shedding in ileum. (**b**) There are villus atrophy, mucosal epithelial degeneration and shedding in jejunum. (**c**) There are villus atrophy, mucosal epithelial degeneration and shedding in duodenum. (**d**) Brain white matter edema: there are areas of vacuolization or reticular looseness (red arrows) between nerve fiber bundles in the white matter, presenting a sponge-like change, and the gaps around blood vessels are enlarged (green arrows).

Histopathology revealed significant villous atrophy throughout the intestines, characterized by shortened, blunted villi, epithelial degeneration, sloughing, and disorganization. Brain examination showed white matter edema, featuring vacuolation, spongiform changes, widened perivascular spaces, and loosened nerve fibers (Figure 4). These findings confirm that the recombinant PSV-ZJ causes severe diarrhea, leading to neurological pathology and ataxia, consistent with CNS involvement.

## 4. Discussion

Previous studies have described PSV as an endemic enteric pathogen causing mild or subclinical diarrheic infections in swine worldwide. Although a UK report noted neurological signs and immunohistochemical detection of PSV in brain tissue, no prior study has definitively demonstrated that PSV can breach the blood–brain barrier (BBB) or cause direct CNS invasion with quantitative viral load confirmation. Moreover, recombination has been hypothesized to drive PSV diversity, but its direct role in conferring novel pathogenic traits such as BBB crossing has never been experimentally established.

The present study presents the first quantitative viral load and histopathological evidence demonstrating that a naturally occurring recombinant PSV strain (PSV-ZJ) has a previously undocumented ability to cross the BBB and induce neurological disease, consistent with CNS involvement. Unlike all previously characterized PSV strains, PSV-ZJ induced 100% morbidity, 20% mortality in the field, and reproduced severe diarrhea, ataxia, cerebral edema, and white matter spongiform changes in piglets. Quantitative qPCR detected high viral loads (up to 10^8.77^ copies/g) in brain tissue, directly confirming BBB breaching-a biological property never before attributed to any sapelovirus. Furthermore, this study demonstrates that an inter-regional recombination event between the Chinese YC2011/2012 strain and the Vietnamese XTND/2019 strain, with breakpoints mapping to the VP2-2A region, contributes to increased clinical severity and neuroinvasiveness compared with earlier PSV isolates. This genomic hotspot encodes capsid proteins and the 2A protease, both critical for cell tropism and immune evasion. The recombination breakpoints located between VP2 and 2A suggest that this genomic region may contribute to the observed virulence phenotype, but direct experimental proof using reverse genetics is required. The study also demonstrates dual respiratory and fecal–oral shedding of a pathogenic PSV and reveals hypothermia as a novel clinical feature. These findings fundamentally change the perception of PSV as a benign enteric virus and highlight recombination as a critical driver of virulence evolution, necessitating urgent surveillance for recombinant strains in global swine trade.

## 5. Conclusions

This study documents the emergence of a novel recombinant PSV strain (PSV-ZJ) isolated in China that exhibits high pathogenicity and the ability to breach the blood–brain barrier and cause neurological disease. These findings highlight a previously underappreciated risk to the global swine industry and emphasize the critical role of recombination in driving the evolution of viral virulence, necessitating enhanced surveillance and the development of targeted preventive strategies.

## Data Availability

Data that support the findings of this study are available from the corresponding author upon reasonable request.

## Supplementary Materials

The following supporting information can be downloaded at: https://www.mdpi.com/article/10.3390/ani16131968/s1.
