# Supplementary material for: Emergence of a Novel Highly Pathogenic Recombinant RNA Virus of Picornaviridae with Blood–Brain Barrier Breaching Capability in China
Source: Animals (Basel). 2026 Jun 25;16(13):1968. doi: 10.3390/ani16131968 (PMC13359431; doi:10.3390/ani16131968)
Supplement: Supplementary file 1 [file animals-16-01968-s001.zip › animals-4347410-supplementary.pdf]

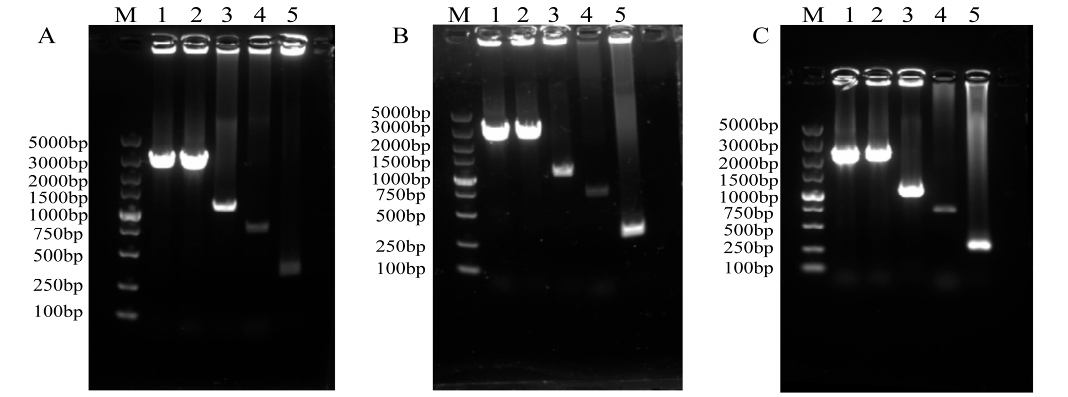

Figure.S1 PSV whole genome sequence amplification results

A: PSV-ZJ-2024 segmented amplification results; B: PSV-FJ-2025 segmented amplification results; C: PSV-SD-2025 segmented amplification results;

M: DL2000 Marker;1~5: Amplification products numbered 1~5

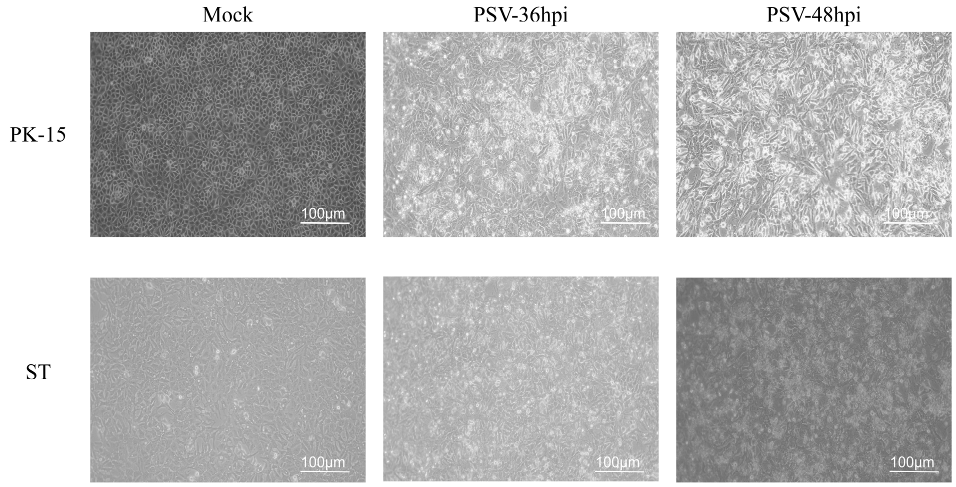

Figure.S2 Morphological observation of isolated strains inoculated into PK-15 cells and ST cells (100×)

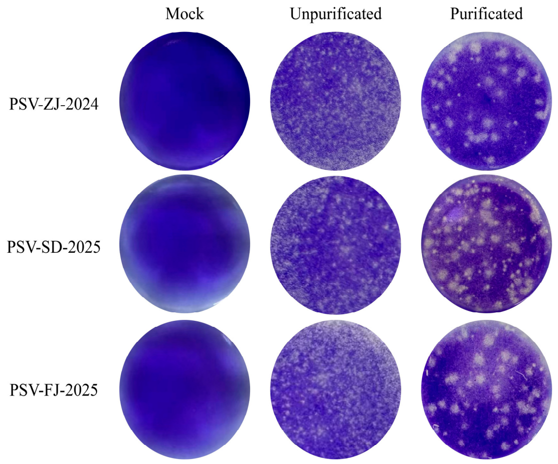

Figure.S3 Purification of plaques from isolated strains

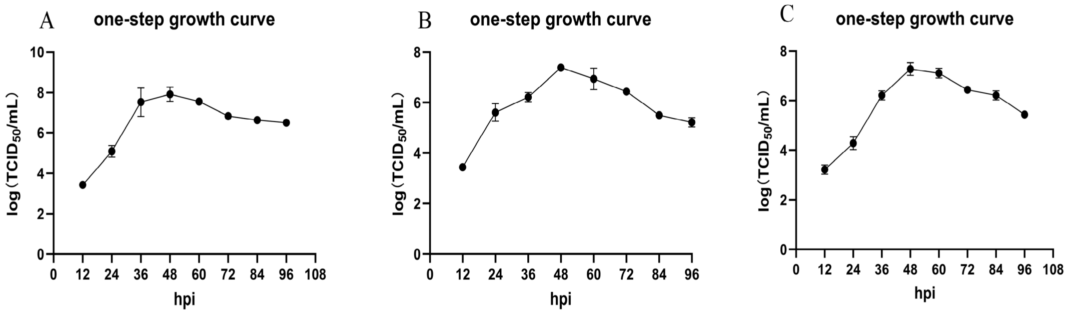

Figure.S4 One-step growth curve of PSV in cells

A: One-step growth curve of the PSV-ZJ-2024 strain; B: One-step growth curve of the PSV-FJ-2025 strain; C: One-step growth curve of the PSV-SD-2025 strain

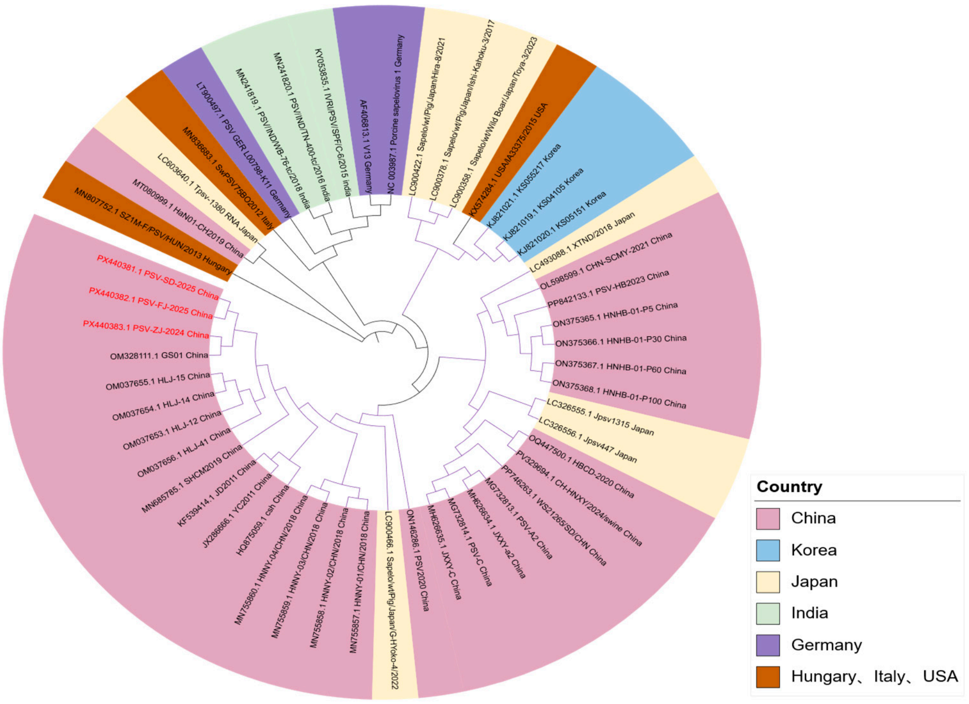

Figure.S5 Phylogenetic analysis based on the complete genome sequence of PSV
